# Early Stage Design Decisions: The Way to Achieve Sustainable Buildings at Lower Costs

**DOI:** 10.1155/2014/365364

**Published:** 2014-01-22

**Authors:** Luís Bragança, Susana M. Vieira, Joana B. Andrade

**Affiliations:** Building Physics & Construction Technology Laboratory, School of Engineering, University of Minho, 4800-058 Guimaraes, Portugal

## Abstract

The construction industry attempts to produce buildings with as lower environmental impact as possible. However, construction activities still greatly affect environment; therefore, it is necessary to consider a sustainable project approach based on its performance. Sustainability is an important issue to consider in design, not only due to environmental concerns but also due to economic and social matters, promoting architectural quality and economic advantages. This paper aims to identify the phases through which a design project should be developed, emphasising the importance and ability of earlier stages to influence sustainability, performance, and life cycle cost. Then, a selection of sustainability key indicators, able to be used at the design conceptual phase and able to start predicting environmental sustainability performance of buildings is presented. The output of this paper aimed to enable designers to compare and evaluate the consequences of different design solutions, based on preliminary data, and facilitate the collaboration between stakeholders and clients and eventually yield a sustainable and high performance building throughout its life cycle.

## 1. Introduction

*“Instead of trying to “force fit” sustainable principles into an existing and often unreceptive manufacturing system, it may be useful to approach the subject from the opposite direction, and consider how functional objects might be designed and manufactured to be compatible with principles of sustainable development” [1].*

Sustainability is an important issue to consider in design, not only due to the environmental concerns but also due to economic and social issues, as they promote architectural quality and have economic advantages [2]. Sustainable design besides contributing to more comfortable and pleasant spaces for living allows economic savings through efficient design while the buildings' environmental footprint is reduced.

**Figure 1:**
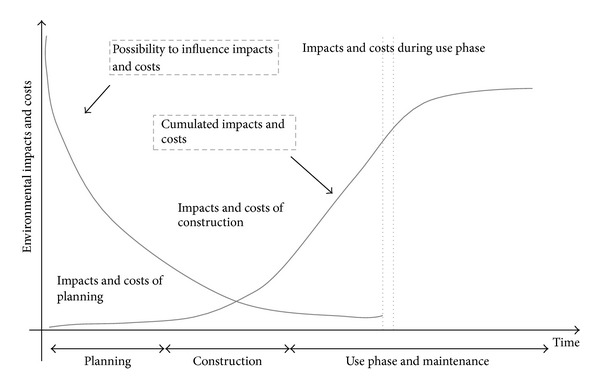
Influence of design decisions on life cycle impacts and costs [35].

The importance of considering sustainability in design stage meets the need for finding long-term solutions that warrant well-being and minimize the needs for natural resources as land use, biodiversity, water, air, and energy. If a project is well planned and sustainable criteria are included in its early approach, the possibility to reduce negative impacts is greater and the cost of criteria implementation is greatly reduced, as illustrated in Figure 1. Improvement of the building's sustainability performance must begin already in the design stage, as the potential of optimisation in project early phases is higher and the impacts of changes of the building and the construction costs are lower.

A building's project obeys general criteria that allow its development on later stages; usually, main criteria respond to functional, economic, social, and time requirements. However, those are not enough to create a consistent base to achieve optimal results for the building. New criteria and approach, that are usually not considered, can bring advantages to the project, favouring improvement of its performance and reducing its final cost [3]. The sooner the project goals are defined and the new criteria are integrated, the more sustainable the building will be.

For the building analysis and sustainability performance prediction in early stage phases of design, several indictors need to be identified and selected. The analysis of the work that has been carried out and published by the research team of the Building Physics and Construction Technology of University of Minho since 2004 [4–15] corroborates this need. Bragança and Mateus publications [15, 16] present previous work from the authors' research team and are in line with the new CEN standards for sustainable construction [17–21], the European energy directive [22], and also major European research projects as perfection [23], SuPerBuildings [24], and Open House [25]. Furthermore, a new set of indicators for early stage design can also be useful for later assessment with the new generation of building sustainability assessment tools.

**Figure 2:**
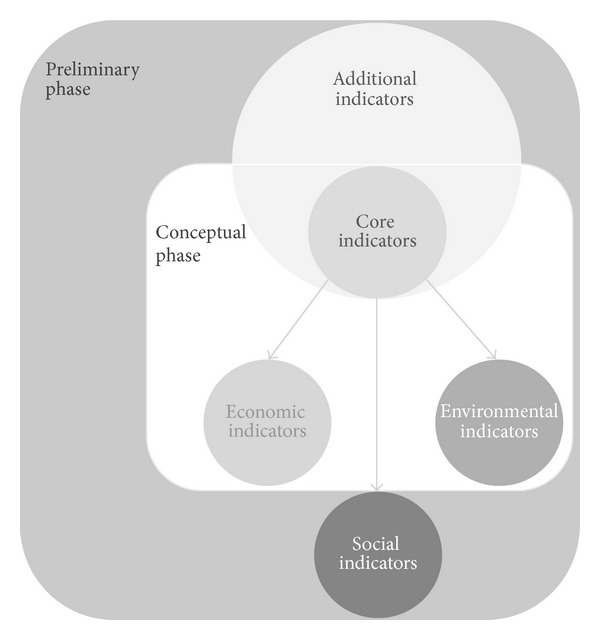
Core indicators and additional indicators.

Two types of indicators can be proposed: core indicators and additional indicators. Core indicators can be used in the conceptual stage, whereas additional indicators can only be used in the next phase, the predesign stage, as illustrated in Figure 2. Core indicators showed to be the best solution for the conceptual stage. The functional unit (square, cubic meter, etc.) to quantify them should be independent from the whole building dimensions, as these later are not available at early design. Moreover, core indicators may be used as a simple and faster assessment, while using both types of indicators—core and additional indicators—gives a more complete and exact evaluation, ensuring sustainability at all fronts of action.

The aim of this paper is to identify the phases through which the design project should go along, emphasising the importance and ability of earlier stages to influence the level of sustainability, performance, and life cycle cost over the project. Afterwards, there is the need to select a set of sustainability indicators and to check its adaptability to the early design. The framework of this paper is divided into two steps: (i) identification and description of project design phases, recognizing the main tasks of each one, and (ii) an analysis of the adequacy and usability of the building sustainability indicators, taking into account the scarce information available at the early design stages. Only core indicators will be described as the scope of this paper focuses on the very beginning of buildings' design—conceptual phase. Additional indicators will be explained in another publication.

The output of this paper aimed to enable designers to compare and predict environmental sustainability performance of different design solutions, based on preliminary data, and facilitate the collaboration between stakeholders. The object of the assessment is the building; it does not include the characteristics of the building site nor its neighbourhood. The scope of the analysis encompasses all stages from material production stage to end-of-life stage.

## 2. Design Phases

A sustainable design needs an integrated design process and a more involved approach than a conventional design process. Ensuring the high quality of design is to ensure an approach based on building performance, an integrated and interdisciplinary project team working through an integrated planning and preparing a project to its best performance. Thus, the design process is crucial because most decisions that will determine building performance in use will be made at this stage.

A building project is developed by a sequence of phases. The concept of design phases is related to a set of consecutive actions that guides the development process. These actions are grouped in stages by their level of priority, shaping each phase of the project. It is important to consider the value of each action/goal/objective, predicting its importance on buildings performance and its influence on the projects final cost in order to implement each one at the adequate moment. Huovila [26] showed that a performance approach is essential to manage life cycle requirements of a building during its conception.

Although different names are given by different authors, the phases of a building project and its goals are generally the same.

The project starts with the definition of its objectives and with the moment where the client meets the project team and exposes the goals for the building. During this initial phase, clients and design team share information seeking to develop the building's concept. The architectural programming is required to define key requirements and constraints towards project quality. Type of architecture and formal and functional aspects must be discussed as well as indoor and outdoor quality desired by the client. Information of the site must be available and if it is not appropriate for construction, elsewhere should be suggested; subjects as room and building functional, environmental, and spatial performance, comfort practices, energy requirements, and so forth should be addressed, as well as concerns on building use, heating, cooling, lighting, ventilation, water, waste, site works, and materials. Additionally, it is at this stage that procurement method, project and sustainability procedures, building design life time, organisational structure, maintenance, project cost, and timescale are dealt with.

The following project step is the implementation of the earlier defined objectives [27–32]. Several publications emphasise the importance of this phase to the performance of the building in its operational phase [28–32]. However, decision making tools are rare [33, 34]. At this phase, all clients' interests and design team members such as architects, engineers, and all needed specialists are involved. This initial phase puts into practice the clients' instructions and exposes the project team proposal; decisions at this early stage are of the utmost importance while project is provisional and open to change.

**Figure 3:**
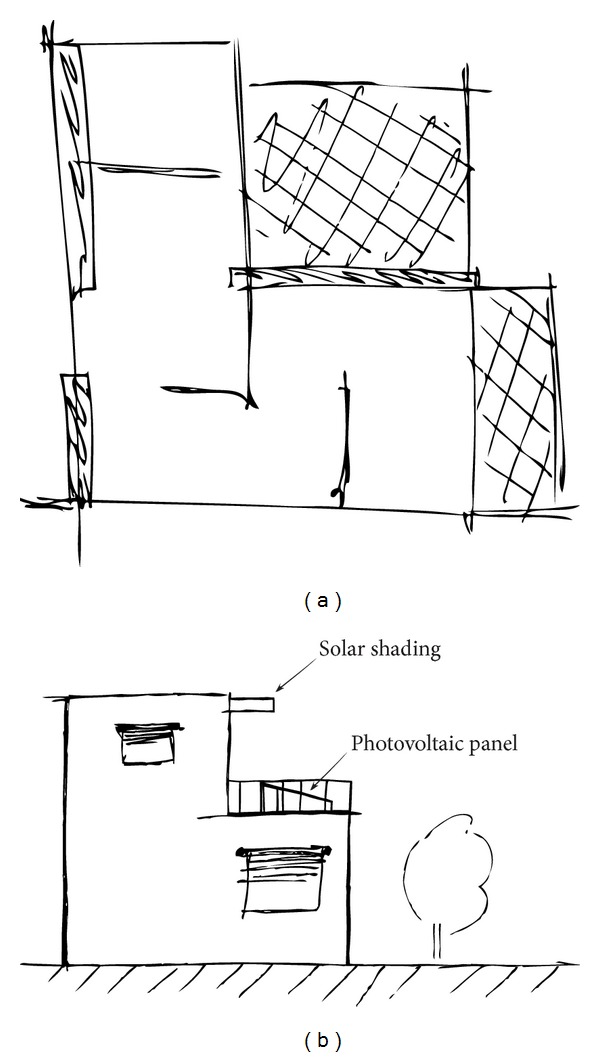
Examples of schematic drawings resulting from the conceptual design phase; (a) spaces first idea/local implementation; (b) first attempt to integrate desired sustainability measures/exterior appearance.

To the scope of this paper, the aforementioned tasks will be grouped into one single design stage—the conceptual stage. Hence, it is hereby understood as the preliminary design phase of the building, in which the overall system configuration is defined, and schematic drawings and layouts will provide an early project configuration, as seen in Figure 3. At this stage, the availability of data is very poor and any assessment has to be based mainly on assumptions. At this stage of design, there are no drawings or any other details about the building. The only information about the building shape is the area of construction and the height of the building. From these elements, all other data need to be estimated. Based on the available input data, the following aspects need to be fulfilled in this stage: the selection of the type of the superstructure of the building; a bill of materials for the structure (estimation); a bill of materials for the envelope (e.g., areas of external and internal walls, area of floors, area of roof, etc.) (estimation).

The next stage of the project begins after the approval of sketch studies; the design team commences the implementation of the working drawings for the construction of the project. Once again different designations are given to this phase—development phase, preproject, basic project [2], and design development [29, 31]. It is also split into two moments, the preliminary project or predesign and the basic project.

At this moment, the general shape of the building is developed through plans, sections, and elevations; the provisional information addressed in earlier phases is confirmed or modified. The actual/chosen solution must be compatible with initial requirements and within the various applicable regulations; the functional relationships between different elements, spaces, and volumes must be examined, as well as the base programming, according to any amendments agreed between the client and the design team.

Type of construction is generally defined and the materials are proposed during the meetings with the clients. Aspects like exterior and interior wall finishing, flooring, plumbing fixtures, hardware design, type of masonry, roofing materials, and so forth shall be decided in this stage. Building equipment as types of windows and doors and their manufacturer, the elevator type and manufacturer, the mechanical system, and electrical fixtures are also to be identified in this phase. This kind of information, when taken together, facilitates an estimate of construction cost. Still, work of every technical specialist must be coordinated; the public authorities must be consulted and initial investigations of comfort and environment should be confirmed.

According to the scope of this work, this phase will be called as predesign phase.

The data available at this stage enables a better definition of the structural system. In this stage, it is expected to have information (drawings) about the plans and elevations of the building. The detail level of the building enables a much more accurate definition of the bill of materials. Based on the available input data, the following aspects need to be fulfilled in this stage: a complete bill of materials for the structure and envelope and the definition of the building orientation.

**Figure 4:**
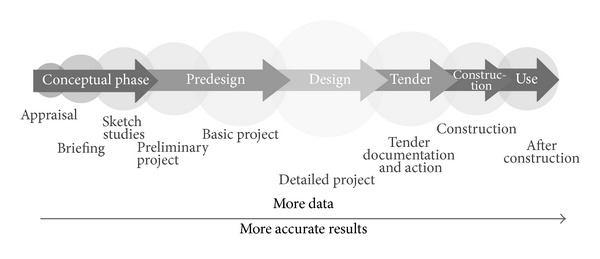
Design stages of a building [36].

Figure 4 summarizes the sequence of the phases, moments, and data improvement of a building project that, from now on, will be used in this work.

Each phase is characterized by a set of key tasks that lead to gathering information needed and to the development of the building architecture and features.

In a conventional design process, these steps can be understood as a linear process, but sequential work routines may be unable to support any adequate design optimization efforts during individual decoupled phases, which of course lead to higher expenditure. In this approach, the architect and the client agree on a design concept, consisting of a general massing schema, orientation, fenestration, and (usually) the general exterior appearance, in addition to basic materials. The structural, building physics, mechanical, and electrical engineers are then asked to implement the design and to suggest appropriate systems. Although this is vastly oversimplified, this kind of process is the one that is followed by the overwhelming majority of general purpose design firm.

On the other hand, a sustainable design needs an integrated design process; it requires the involvement of the whole design team and the iteration between phases. The design team must maintain a high level of communication throughout the design process and must work well together to resolve all issues and concerns on the project. Accordingly, the attitude of the design team is critical and their members must be able to establish a collaborative framework for the project.

Although conceptual and predesign phases have been defined, in this paper, only conceptual phase will be considered for the inclusion of sustainable concerns. It is considered to be the most crucial phase, as less data is available and the possibilities to design and innovate are greater.

## 3. Selection of Indicators

As mentioned previously in this paper, core indicators aim to predict the building's sustainability performance at conceptual phase, where the data available is scarce. Environmental impacts, energy, and life cycle costs related indicators were considered to be those which have a major influence on sustainability and are able to be assessed at conceptual design phase.

### 3.1. Environmental Impacts

Environmental impacts category is composed of one single indicator—*aggregated value of environmental impact*—which in turn gathers the seven subindicators proposed in EN 15643-2:2011, listed in Table 1. These sub-indicators are evaluated based on characterization factors and input flows.

Normal life cycle impact estimator software (as SimaPro or GaBi) can be used to estimate these values. As in the project conceptual stage the exact amount or construction technology to be used is under determination, and a database of the buildings' envelope elements and its environmental life cycle impact is needed. A database with this purpose has already been published by Bragança and Mateus [37]. It presents environmental life cycle data impact for several building elements and materials, aiming to support design decision making towards greater environmental performance of their buildings. Based on a comparison, the designers will be able to determine the solutions with less environmental impact or resource use.

### 3.2. Energy

*Total primary energy demand* describes the energy consumption predicted to the operational phase, summarized in Table 2. Guidelines for energy consumption impose the improvement of energy efficiency and the reduction of consumption [38, 39]. For that reason, the total demand for primary energy should be minimized and the share of renewable energy should be maximized while reducing the share of nonrenewable energy during the building's life cycle. This indicator accounts for estimation of (i) energy use for space heating, (ii) energy use for space cooling, (iii) energy use for domestic hot water production, and (iv) other energy uses. To assess energy performance, an algorithm to compute operational energy at early design stage needs to be used. National, regional, or local codes of practice for thermal behaviour and energy efficiency are most often simple calculation procedures that can be easily used to estimate the energy consumption at the operational phase.

### 3.3. Life Cycle Cost

The indicators describing life cycle cost are listed in Table 3. Construction costs are all the costs related to each process needed to build the building. This indicator includes (i) the cost of material acquisition and transportation, (ii) the cost of construction equipment, and (iii) the cost of manpower. Most of these costs are usually calculated based on the bill of materials and unit costs provided in the project. These costs usually occur in the first or second years of the building life cycle. However, due to the long time period of analysis, it may be assumed that they occur in the first year, the base year, of the building life cycle.

Maintenance costs include all costs occurring over the service life of the building, in order to keep it according to the required functional conditions. End-of-life costs refer to the end-of-life activities such as the total or partial demolition of the building and the removal of the demolition waste to its final destination. These costs may be estimated based on scenarios and best practices.

Thus, the authors will take advantage of their team previous work [15, 16] and the work done by Fuller and Petersen [40] and use their approach to implement costs quantification.

### 3.4. Summary of Selected Core Indicators

In order to ease understanding, Table 4 summarizes the core indicators proposed. As seen above, eleven indicators to be included in the initial phases of design were selected. The first seven regard environmental impact categories, such as global warming or abiotic resource depletion, which can be easily estimated through a database or a catalogue of buildings' elements LCA. The eighth indicator considers primary energy demand. Although it may look difficult to assess this indicator at an early design stage, the use of codes of practice for thermal behaviour and energy efficiency allows simple assessments. At last, three cost related indicators were considered. All three represent an estimation of the buildings life cycle costs, which represent a major aspect to take into account since early stages. Typically, stakeholders only consider initial costs neglecting all the other attitudes that may lead to more costly buildings when looking to its entire life.

## 4. Conclusions

The aim of this paper is to determine which sustainable indicators can be assessed in the initial phases of a design project.

For that, an initial study was carried out to clarify the contents of the early stages of design of a building. It was concluded that, although different names are given, most of the available literature identifies the same stages. From the several designations and stages, the following were analysed in this work:

- conceptual phase: it begins when the client meets the design team and the objectives of the project are defined. It represents a preliminary design phase of the building, in which the overall system configuration is defined and schematic drawings and layouts will provide an early project configuration, type of architecture, and formal and functional aspects. It lacks specific data;
- predesign phase: it starts with the implementation of the working drawings; the general shape of the building is developed through plans, sections, and elevations; the provisional information addressed in the conceptual phase is confirmed or modified.

Several methodologies, standards, and research projects were analysed to determine the final set of key indicators that should be considered in this methodology, supporting assessing and management of project process during early design phases.

The conceptual phase deals with fuzzy data and often lacks information, which makes it impossible to address several indicators, especially the ones related to the social and functional aspects of the building.

Taking this into consideration, two groups of indicators were settled: (i) core indicators and (ii) additional indicators. Core indicators can be used in the conceptual stage, whereas additional indicators can only be used in the latter stages (predesign).

The core indicators regard the aspects that can be addressed under the little information available at the conceptual phase. On the other hand, additional indicators compile all the other indicators. In this sense, core indicators consist in environmental impacts, energy consumption, and costs, whose data can be obtained from databases of buildings' elements, simple estimation of the operational energy demands, and databases of construction solutions costs.

In conclusion, from this study, it is essential to consider sustainability concerns since the first stages of design project so as to assure greater performances. Environmental impacts and life cycle costs are most likely to be easily considered during conceptual stage, as they require less information from the building, while social concerns need to deal with several specifications of the in order to be quantified.

## Figures and Tables

**Table 1:** Subindicators describing environmental impact indicator.

Indicator	Unit
Global warming potential, GWP	kg CO_2_ equiv
Depletion potential of the stratospheric ozone layer, ODP	kg CFC 11 equiv
Acidification potential of land and water; AP	kg SO^2−^ equiv
Eutrophication potential, EP	kg (PO_4_)^3−^ equiv
Formation potential of tropospheric ozone photochemical oxidants, POCP	kg ethene equiv
Abiotic resource depletion potential for elements; ADP_elements	kg Sb equiv
Abiotic resource depletion potential of fossil fuels ADP_fossil fuels	MJ

**Table 2:** Indicators describing energy impacts.

Indicator	Unit
Total primary energy demands and share of renewable and nonrenewable primary energy resources (in operation phase)	kWh/m^2^·year

**Table 3:** Indicators describing life cycle costs.

Indicator	Unit
Construction costs	*€*/m^2^
Operation costs	*€*/m^2^
End-of-life costs	*€*/m^2^

**Table 4:** List of selected indicators for conceptual phase.

Environmental indicators	
Environmental impact	(1) Global warming potential
	(2) Depletion potential of the stratospheric ozone layer
	(3) Acidification potential of land and water
	(4) Eutrophication potential
	(5) Formation potential of tropospheric ozone photochemical oxidants
	(6) Abiotic resource depletion potential for elements
	(7) Abiotic resource depletion potential of fossil fuels

Energy	(8) Total primary energy demand

Economic indicators	

Life cycle costs	(9) Construction costs
	(10) Operation costs
	(11) End-of-life costs
